# Direct Support Professionals’ Perspectives on Using Technology to Help Support Adults With Autism Spectrum Disorder: Mixed Methods Study

**DOI:** 10.2196/40722

**Published:** 2023-04-25

**Authors:** Christina A Simmons, Abigail E Moretti, Andrea F Lobo, Patrice D Tremoulet

**Affiliations:** 1 Department of Psychology Rowan University Glassboro, NJ United States; 2 Department of Computer Science Rowan University Glassboro, NJ United States

**Keywords:** technology, data collection, documentation, direct support professionals, autism, mobile phone

## Abstract

**Background:**

Documentation is a critical responsibility for direct support professionals (DSPs) who work with adults with autism spectrum disorder (ASD); however, it contributes significantly to their workload. Targeted efforts must be made to mitigate the burden of necessary data collection and documentation, which contributes to high DSP turnover rates and poor job satisfaction.

**Objective:**

This mixed methods study aimed to explore how technology could assist DSPs who work with adults with ASD and prioritize aspects of technology that would be most useful for future development efforts.

**Methods:**

In the first study, 15 DSPs who worked with adults with ASD participated in 1 of the 3 online focus groups. The topics included daily tasks, factors that would influence the adoption of technology, and how DSPs would like to interact with technologies to provide information about their clients. Responses were thematically analyzed across focus groups and ranked by salience. In the second study, 153 DSPs across the United States rated the usefulness of technology features and data entry methods and provided qualitative responses on their concerns regarding the use of technology for data collection and documentation. Quantitative responses were ranked based on their usefulness across participants, and rank-order correlations were calculated between different work settings and age groups. The qualitative responses were thematically analyzed.

**Results:**

In study 1, participants described difficulties with paper-and-pencil data collection, noted benefits and concerns about using technology instead, identified benefits and concerns about particular technology features, and specified work-environment factors that impact data collection. In study 2, participants rated multiple features of technology as useful, with the highest usefulness percentages endorsed for task views (ie, by shift, client, and DSP), logging completed tasks, and setting reminders for specific tasks. Participants also rated most data entry methods (eg, typing on a phone or tablet, typing on a keyboard, and choosing from options on a touch screen) as useful. Rank-order correlations indicated that the usefulness of technology features and data entry methods differed across work settings and age groups. Across both studies, DSPs cited some concerns with technology, such as confidentiality, reliability and accuracy, complexity and efficiency, and data loss from technology failure.

**Conclusions:**

Understanding the challenges faced by DSPs who work with adults with ASD, and their thoughts about using technology to meet those challenges, represents an essential first step toward developing technology solutions that can increase DSPs’ effectiveness and job satisfaction. The survey results indicate that technology innovations should incorporate multiple features to account for different needs across DSPs, settings, and age groups. Future research should explore barriers to adopting data collection and documentation tools and elicit input from agency directors, families, and others interested in reviewing data about adults with ASD.

## Introduction

### Background

Many adults with autism spectrum disorder (ASD) require assistance from direct support professionals (DSPs) to live and participate successfully in their communities. These DSPs provide daily support ranging from basic care needs, such as grooming, hygiene, dressing, and eating, to assistance in living independently and engaging in the community [1]. Responsibilities of DSPs are multifaceted, including shaping new skills; following treatment plans to decrease aberrant behaviors; transporting and assisting with vocational, recreational, and therapeutic activities; administering medication; communicating with diverse medical professionals; and documenting care administered and progress toward behavioral goals [2,3].

Throughout the United States, the demand for DSPs has far exceeded the supply [4-6]. In 2015, the estimated number of DSPs in the United States was 3.38 million [2]. By 2024, researchers anticipate a 26% increase in the need for personal care aides and a 38% increase in the need for home care aides [3]. High turnover is common, especially in DSPs working with individuals who present with behavioral challenges [7]. Before the COVID-19 pandemic, staffing shortages, overload, and stress contributed to a 20% vacancy rate and 44% turnover rate among DSPs [8,9]; shortages and turnover have only increased since then [10,11]. Alarming statistics indicate that 55% of DSPs leave within their first year of employment, with approximately 35% leaving within the first 6 months [3]. The most frequently reported reason for high turnover among DSPs is low salary. Other contributing factors include burnout from high stress, isolation, high likelihood of injury, insufficient supervision, lack of career advancement opportunities, and inadequate training [3,7].

A critical DSP responsibility is to record client behavior. This information is needed to evaluate progress with specific programming, assist in treatment decision-making, inform the client’s family and funding agencies, and communicate with DSPs working with a particular client [12]. Documentation requirements contribute significantly to DSP overload and stress, and turnover tends to increase workload, as DSPs left behind must generate detailed documentation to help orient new DSPs to their clients’ needs. Moreover, overworked DSPs may struggle to find time to document and share all relevant data about their clients during shift changes, in addition to completing other tasks, such as supporting medical and behavioral health needs or teaching workplace, social, and self-help skills. These documentation challenges leave adults with ASD, who rely upon DSPs, susceptible to medical errors and inadequate support, which can contribute to physical or emotional harm [7].

One way to address these documentation challenges is through the implementation of digital technology systems. Generally, technology refers to the use of various approaches (eg, algorithms and software) for data processing, with digital technology encompassing internet-based data processing through computers, mobile devices (eg, smartphones and tablets), and apps [13,14]. Although existing digital technology has the potential to increase documentation efficiency, the introduction of electronic health records (EHRs) into health care systems has demonstrated that it is critical that digital technology supporting documentation be designed to make it as easy as possible to input the required information. Several studies indicate that EHRs make it easier for health care providers to organize and access information, including physicians [15], residential staff in aged care facilities [16], and residential staff in agencies serving individuals with intellectual disabilities [17]. However, unfortunately, EHRs have actually decreased physician efficiency and hindered workflow because of the difficulty in recording patient data [18]. Providers often need to remember patient information or document it via paper and pencil until they are able to access a computer to enter it into the EHR [19], which can increase the likelihood of human error and inaccurate data [20]. Furthermore, physicians reported that usability issues, slow system response times, information overload, and difficulties finding information are associated with stress, burnout, and increased workload [18,21-25]. Because of the increased time spent on EHR-related tasks, physicians report working longer hours and completing documentation outside of work, which further contributes to stress and burnout [23-25]. A systematic review in 2022 indicated that over the past 5 years, EHR documentation time contributed to the symptoms of burnout, including cognitive and physical fatigue, perceived lack of autonomy, and poor work-life balance [18].

To address the limitations of EHR systems, researchers have begun to examine mobile technologies that facilitate in-the-moment documentation to improve both data accuracy and efficiency. The use of such mobile technology to support providers in collecting in-the-moment data has been demonstrated in the fields of special education and applied behavior analysis (ABA). Research conducted with special education teachers serving students with behavioral challenges explored the use of automated video and audio capture, with selective user archiving via a button press. The results indicated that the CareLog system, which offered these features, made it easier for teachers to collect in-the-moment data, and it was preferred to pencil-and-paper data collection, despite needing to watch and analyze video clips at a later time [26].

Furthermore, data collection software has been created specifically for collecting behavioral data during ABA services, which permits providers to enter in-the-moment data via a device (ie, mobile phone, tablet, and computer), review synthesized data, and track client progress [27]. With the ability to simplify data collection and analysis, aggregate client data, and create automated graphs, these web-based and mobile apps improve the documentation efficiency for ABA providers and increase the accuracy of data collection, both of which can contribute to improved care for clients.

Mobile technology has also been explored for in-the-moment data collection conducted by caregivers. Researchers explored the utility of the My JAKE mobile app, part of the Janssen Autism Knowledge Engine, for caregivers collecting in-the-moment, observational data on their child’s sleep quality, affect, and other behaviors. The app included alerts to remind caregivers to submit daily reports and permitted clinical teams to observe data in real time via the My JAKE dashboard. Caregivers consistently submitted daily reports and perceived the mobile app to be easy to use, suggesting that the My JAKE mobile app has important implications for clinical practice for children with ASD, including the ability of clinical teams to observe behavioral data while the child is at home, which could inform interventions [28].

Despite findings indicating that mobile and web-based technology can increase efficiency for in-the-moment data collection, most tools are targeted toward providers working with children, and little research has been conducted on the use of technology for in-the-moment documentation for DSPs working with adults with ASD. Thus, the utility of data collection systems via mobile technology for DSPs working with adults with ASD should be explored, as the work settings and data collection needs differ. The benefits of a digital technology support system for DSPs could include (1) reducing the DSP workload; (2) increasing the time DSPs spend interacting with adults with ASD; (3) enabling DSPs to provide more consistent and appropriate support; (4) increasing DSP job satisfaction; (5) improving medical and behavioral support for adults with ASD; and (6) providing a foundation for technology use that increases independence in adults with ASD, which ultimately leads to improved quality of life. Digital documentation could facilitate timely access to information about adults with ASD for diverse stakeholders who help support those adults, such as family members, therapists, behavior analysts, health care providers, and DSPs.

### Objectives

Despite the potential benefits of mobile technologies for reducing the burden of documentation, there is a dearth of research examining the utility of such technology for DSPs working with adults with ASD, highlighting a critical gap in the literature that this study addresses. This 2-part mixed methods study aimed to determine and prioritize how digital technology could help DSPs more easily produce, collect, present, and share data about their clients. Although it is widely known that DSPs significantly impact their clients’ quality of life, most research aiming to improve the quality of life of individuals with ASD focuses on interventions for these individuals. Owing to the unique aspects of a DSP’s job responsibilities and employment settings, it is necessary to use user-centered design practices to ensure that technological solutions will be useful, usable, and acceptable to DSPs. Through focus groups with DSPs, we captured their perspectives on current data collection and documentation needs, and their ideas on how digital technology could be applied to improve their ability to support adults with ASD. A nationally disseminated survey evaluated the usefulness of different features of technology and data entry methods across DSPs working in different settings for adults with ASD. Our summary of DSP thoughts about using technology on the job, quantitative rankings of the usefulness of potential technology innovations, and cited concerns can inform the development of technology that supports DSPs who work with adults with ASD.

## Methods

### Ethics Approval

This study complied with the American Psychological Association Code of Ethics and was approved by the Institutional Review Board of (Rowan University: Pro2020-001085). Informed consent was obtained via videocall from each participant. To maintain the privacy and confidentiality of participants, focus group data were deidentified, and survey data were collected anonymously. The focus group participants were compensated for their participation with a US $75 electronic gift card and survey participants could enter into a drawing for 2 US $75 electronic gift cards by providing their contact information via a separate link not associated with their survey responses.

### Focus Groups

#### Overview

A total of 3 online focus groups were conducted using Cisco WebEx v.40.11.4.15, a Health Insurance Portability and Accountability Act (HIPAA)–compliant videoconferencing platform. Focus groups were conducted until saturation of themes was achieved, wherein no new themes or subthemes emerged from the data [29], with a minimum of 3 focus groups. The decision to conduct at least 3 focus groups is supported by the literature on the number of focus groups required to identify prevalent themes [30]. The focus groups included 4 to 6 participants (focus group 1, n=6; focus group 2, n=4; focus group 3, n=5), consistent with the literature on the benefits of smaller-sized online focus groups [31]. Online focus groups provide several advantages, including ease of recruitment of a difficult-to-reach population, increased accessibility, decreased cost and use of resources, and increased comfort for participants [32-34]. See Table 1 for the demographic information of the participants.

**Table 1:** Focus group participant demographics.

Demographic variable			Value, n (%)
**Settings (n=15)**			
	Large agency day program	5 (33)	
	Private home with single resident	4 (27)	
	Large agency residential	3 (20)	
	Midsized agency day program	2 (13)	
	Family home	1 (7)	
**Number of clients served at a time (n=15)**			
	1	4 (27)	
	2	1 (7)	
	>3	10 (67)	
**Number of years in field (n=14)^a^**			
	<1	2 (14)	
	1-3	3 (21)	
	3-5	1 (7)	
	5-7	6 (43)	
	7-10	0 (0)	
	>10	2 (14)	

^a^One participant joined the last focus group late and thus did not participate in the introductions where others shared how many years of experience they had worked with adults with autism spectrum disorder.

A total of 15 individuals who worked with adults with ASD as DSPs were recruited through targeted emails and a recruitment flier shared via email, in person, social media, and through direct mail with local organizations that provide services to adults with ASD in a densely populated northeastern state.

We used a standard focus group methodology [35] to elicit perspectives from the DSPs on data collection, information reporting, and the potential use of technology in their daily responsibilities. All focus groups were facilitated by PT, assisted by trained researchers (CS and AM), and lasted a mean of 89.3 (SD 20.3; range 73-112) minutes. The facilitator managed the discussions to ensure that the conversation did not bias the participants.

Guided focus group topics included the following:

1. What tasks do DSPs perform frequently, and which could be at least partially automated and assisted by technology?
2. Are they aware of technologies that can help them with data collection and documentation?
3. What factors would influence DSP’s adoption of technology to assist with data collection and documentation?
4. How and when would DSPs like to provide information about their clients?
5. What sort of privacy safeguards would they expect when using technology to assist in data collection and documentation?
6. What factors should influence how digital information is requested or presented?
7. How useful do they believe a technology solution could be?

#### Data Preparation and Analysis

Data from voice recordings of focus groups were transcribed using the automated transcription feature on Webex, manually reviewed by researchers alongside audio recordings for errors and clarification, and cleansed of identifying information. All references that could link data to the participant’s identity were removed or altered to preserve confidentiality (eg, names were changed to participant numbers). PT reviewed the final focus group transcripts against the recorded conversations to ensure accuracy and completeness. Minor corrections were made to the transcription, as necessary. The trained data coder (AM) entered the data into spreadsheets to facilitate storage, coding, retrieval, comparison, and linking.

All the guiding question topics were addressed in each of the focus group discussions. Using the constant comparative method of qualitative data analysis [36,37], data were analyzed inductively to identify categories that emerged from participant narratives rather than to conduct provisional hypothesis testing. Salient themes were established using 6 phases of thematic analysis in psychology [29]. Specifically, researchers reviewed the transcript data to identify categories that emerged from participant narratives, combined categories into relevant themes, and adjusted theme definitions as new categories emerged. Saturation was reached by the third focus group, as no new major themes or subthemes emerged in participant responses to questions from the moderator guide. Researchers then organized the identified themes into a comprehensive codebook that included operational definitions of each theme, subthemes to specify theme content, and specific exclusionary criteria. The trained data coder then reviewed the transcript data and coded participant narratives into established themes to examine the frequency with which each theme was discussed. Transcripts did not capture head nods or other nonverbal cues, indicating agreement with other participants’ comments; thus, only verbal responses were coded into the established themes. Interrater reliability was determined by having 2 members of our research team trained in qualitative data analysis independently code 33% of the focus group transcripts using the team-developed codebook. The interrater reliability was 90%.

#### Ranking and Scoring

To rank the salience of themes as one piece of information when considering DSPs needs, the total count of each theme was summed across participants in each focus group and across all 3 focus groups [38,39]. Each example or explanation provided by the participants was counted as an occurrence. A separate occurrence was documented once the participant provided a different explanation or another participant responded. Revisiting a previous example or explanation was counted as a separate occurrence. Although the key information gleaned through qualitative data analysis is the distinct themes that emerge from the data, recent research using the constant comparative method of qualitative data analysis has presented the salience of themes using this methodology as an accompanying piece of information [38].

Conducting focus groups with DSPs is an important first step in identifying the needs and ways to support DSPs. To build upon the focus group themes and identify the technology features that would have the greatest positive impact on DSP workload and job satisfaction, we developed a web-based survey that obtained input from a larger sample of DSPs.

### Web-Based Survey

#### Overview

A power analysis indicated that a sample size of 147 was required to detect a large effect at a significance criterion of α=.5. Survey participants were recruited through emails sent to agencies serving adults with ASD and social media recruitment posts to groups and forums frequented by DSPs. A total of 728 agencies were contacted across 33 states, representing all the geographic regions of the United States. Individuals were eligible to participate if they were aged ≥18 years and had worked or were currently working with one or more adults with ASD as a DSP in the United States. A total of 179 participants participated in this survey. After excluding participants who did not consent to the survey (n=2), lived outside the United States (n=1), did not answer any open-ended questions (n=11), or did not complete any Likert-scale items (n=12), a total of 153 participants were included in the final sample.

#### Survey Completion

A web-based survey administered by Qualtrics CoreXM (Qualtrics) provided additional DSP perspectives on the use of digital technology to support their work. Questions included demographics (eg, sex, race, and native language), work specific information (eg, job settings and responsibilities), ways technology could be useful for DSPs for task completion and documentation (eg, viewing tasks for current shift), and data entry (eg, typing on a phone or tablet). Questions regarding the usefulness of technology were organized into 2 matrices (ie, features of technology and methods of data entry) with 4-point Likert-scale responses: Not Useful (1), Maybe Useful (2), Probably Useful (3), and Definitely Useful (4). Finally, an open-ended question invited participants to share concerns about any aspect of technology for data collection and documentation. See Table 2 for the demographic information. The participants had a mean age of 36 years (SD 9; range 19-68 years; median 35 years) and had worked in 41 different states, with the greatest representation from New Jersey, Pennsylvania, Texas, and Illinois. Participants had worked with adults with disabilities for a median of 4 years (range, 4 months-45 years). The survey took a median of 2.2 minutes to complete (range 1 minute 34 seconds-1 hour 40 minutes).

**Table 2:** Demographic variables of survey participants. Participants could choose “Other” and provide a qualitative response if other survey options did not adequately reflect their experiences (N=153).

Demographic variables			Value, n (%)
**Sex**			
	Female	98 (64.1)	
	Male	52 (34)	
	Other	3 (2)	
**Race**			
	White	83 (54.2)	
	Asian	34 (22.2)	
	Black or African American	30 (19.6)	
	Native American or indigenous	1 (0.7)	
	Native Hawaiian	1 (0.7)	
	Other	4 (2.6)	
**Native language**			
	English	125 (81.7)	
	Spanish	26 (17)	
	Other	2 (1.3)	
**Level of education**			
	Less than high school	0 (0)	
	High school or GED^a^	23 (15)	
	High school + certifications	20 (13.1)	
	Some college	39 (25.5)	
	Finished college	46 (30.1)	
	Some or finished graduate school	25 (16.3)	
**Work setting**			
	Community	58 (37.9)	
	Residential facility	55 (35.9)	
	Home	46 (30.1)	
	Day program	37 (24.2)	
	School	24 (15.7)	
	Clinic	22 (14.4)	
	Other^b^	4 (2.6)	
**Job responsibilities**			
	Promote healthy behaviors	79 (51.6)	
	Promote social skills	77 (50.3)	
	Reduce undesirable behaviors	74 (48.4)	
	Teach daily living skills	70 (45.8)	
	Assist with activities of daily living	65 (42.5)	
	Teach vocational skills	50 (32.7)	
	Train staff	49 (32)	
	Cleaning and housekeeping	47 (30.7)	
	Assist with feeding	42 (27.5)	
	Train caregivers	33 (21.6)	
	Teach academic skills	30 (19.6)	
	Other^c^	10 (6.5)	
	Treat addiction	5 (3.2)	

^a^GED: general equivalency degree.

^b^Other work settings include vocational settings.

^c^Other job responsibilities include administering medication, administering medical care, transportation, shopping, and outdoor chores.

#### Data Preparation and Analysis

Data were extracted from Qualtrics and cleaned to remove missing data. Descriptive statistics were calculated across demographic variables (ie, mean, median, and range) and Likert-scale responses (ie, percentage). Because the goal of this exploratory research was to understand how technology could benefit DSPs, “Definitely Useful” and “Probably Useful” responses were combined to create a “Useful” category. Responses were ranked by the overall percentage in the Useful category. Data were separated by work setting (ie, community, residential, day program, school, and clinic) and median age (ie, ≤35 years and >35 years) to distinguish between older and younger participants in our sample. Spearman rank-order correlations were calculated, and the probability computed for a 1-tailed test at the level α=.05, determined a priori.

Open-ended responses were thematically analyzed using the constant comparative method of qualitative data analysis [36,37] to identify categories that emerged from participant responses. The frequency of responses in each category was summed to determine the salience of themes across participants. Interrater reliability was determined by having 2 members of our research team trained in qualitative data analysis independently code open-ended responses. We divided agreements by agreements plus disagreements, and converted them into percentages. The interrater reliability was 98.4%.

## Results

Throughout this section, themes and subthemes are listed in order of salience (ie, the greatest frequency of mention). For focus groups, multiple mentions from the same participant were counted as multiple instances.

### Focus Groups

#### Overview

The focus group participants described their primary roles and responsibilities as keeping their clients and others safe, assisting clients with completing activities of daily living, and creating meaningful opportunities for engagement. In addition, the participants indicated that they were responsible for data collection and documentation as a secondary priority to direct client care. All participants indicated that technology could help, in some capacity, with their daily work.

Participants in each focus group mentioned all themes. No new subthemes emerged after the second focus group. Table 3 shows the frequency of themes across focus groups. The mean rank-order correlation between overall theme rankings and individual focus group rankings was 0.6 (range 0.5-0.8), indicating a moderate positive correlation. Table 4 summarizes the participants’ perspectives on the use of technology to support their work.

**Table 3:** Themes by focus group (FG).

Theme	Overall *f*^a^	FG 1 *f*	FG 2 *f*	FG 3 *f*
1. Benefits of technology for data collection	98	33	33	32
2. Useful features of technology	66	16	11	39
3. Concerns with features of technology	54	9	10	35
4. Concerns with technology for data collection	51	18	20	13
5. Work-environment impact on data collection	34	21	9	4
6. Pencil-and-paper data collection difficulties	30	7	16	7

^a^*f*: frequency.

**Table 4:** Technology or features of technology considered by direct support professionals in focus groups.

Technology or feature	Potential use	Potential concerns and considerations
Video recording	Data review Different vantage point Supervisor feedback	Client confidentiality Staff confidentiality Portability Remote playback option to easily access specific recording Time to review recordings
Voice capture and activation	Data collection Documentation Hands-off	Inaccurate information capture Sensitivity to different speakers Difficulty registering in loud environment Client manipulation Client sensitivity to information stated Need to review for accuracy
Reminders and alerts	Reminders of timely tasks that must be completed Alerts for next shift or supervisor to review Alert to seek assistance Alerts for clients could promote independence	Alert fatigue Management of simultaneous alerts for multiple clients Clients aware of other client’s information
Information flags	Notice of important client information to review	Time to review information
Automatic graphing ability	Efficient data review and progress monitoring	None
Auto population of reports	Automatic synthesis of data and documentation into required reports	Need for qualitative data entry to explain contextual variables
Portable device	Data collection in community or throughout client’s daily activities	Device not functioning properly or as intended Need to use personal device
Hands-free device	Wearable device for portable unobtrusive data collection	Should have breakaway straps to remove in event of client aggression
Continuous data collection and documentation	Documentation throughout a shift rather than recalling all information at end of shift	Available time
Button press information capture	Quick data collection that allows eyes on client	Client manipulation
Offline technology	Data collection when in community or without internet connection	Need to communicate with supervisors during an incident
Digital client information database	Easy access to client information and protocols	Time to review information
Standardized data entry	Efficient and consistent documentation across providers	All relevant information captured
Communication across staff	Easy way to enter and send important messages Communication without client's awareness Communication with supervisor during an incident	Continuous communication even when off shift if sent to personal device HIPAA^a^ compliance if using personal device

^a^HIPAA: Health Insurance Portability and Accountability Act.

#### Theme 1. Benefits of Technology for Data Collection and Documentation

Participants reported that technology presents potential benefits for data collection and documentation, an essential, albeit nonpreferred, component of their daily responsibilities. Participants described using technology as easier and more flexible than paper and pencil (f=37). For example, 1 participant shared “If I had an iPad that had an application with the notes, I could just like, pop up and walk around with that. My life would be easier.” Another participant shared, “I’ve done it like paper and pencil and then I’ve done it with a tablet and the tablet is faster and easier.” They noted the ability to communicate easily with other staff (f=25) and having data organized in one location (f=18) as benefits. For instance, one participant described the benefit of shift notes as follows:

...just to know about how the individual’s day was: what they ate, their mood, if they had any bowel movements, food intake, community outings, stuff like that...I’ve noticed that has been helpful, as far as communication purposes, because I know when I come in 7:00 am, um, I know how my individual slept for the night...

They also viewed using a personal mobile device to collect data favorably (f=8), noting that they could have it readily accessible, would be solely responsible for the device, and would not need to manage another item. They suggested that data collected via technology may be more accurate (f=5), particularly for data collected while simultaneously completing multiple tasks or by automating data collection across staff. Finally, they indicated that the use of technology may be unobtrusive, as many clients have their own devices and are accustomed to having devices around them (f=5).

#### Theme 2. Useful Features of Technology

Participants reported several specific features of the technology that could be useful in their work. They described the utility of using cameras and audio recordings to review behaviors or protocols (f=30). Multiple DSPs in this study suggested that video technology could facilitate capturing potential antecedents to challenging behavior, for example:

I think [cameras are] actually pretty necessary to see...[w]hat led up to that? and We might think some things are just, you know, jumping out of the blue, but there may actually be behaviors that the video recorder may see or be able to pick up. They might actually lend itself to being able to kind of identify what was happening.

Another participant described the benefit of recordings to inform feedback and for personal growth

I’m able to say, “Hey, can you look back and tell me what I could have done better or what I could have done differently?”

Participants also described the benefits of reminders or alerts (f=19) for the many tasks they needed to complete, such as medication administration or documentation. One participant described how such reminders could facilitate smooth transition between DSPs,

If you’re understaffed, or if your shift ended and somebody else is coming in, it gives reminders or like, a list of things that still need to be done.

With regard to alerts, participants described how wearable technology might be used to capture data and flag segments for review at a later time. Other useful features suggested by participants included the use of voice to capture information (f=12), particularly when DSPs’ hands are occupied with other tasks and paper-and-pencil data collection may be infeasible, and automatic graphing abilities (f=5) to generate visual representations of data for immediate visual analysis.

#### Theme 3. Concerns With Features of Technology

Although participants indicated that technology could be useful, they also expressed some reservations about specific features. Participants were concerned about the accuracy of voice-captured information (f=21), particularly sensitivity to different accents of a diverse population of DSPs. They also shared concerns about clients interfering with, imitating, or being upset by voice-captured data (f=11). For example:

I’m just envisioning, like me saying...“Alexa, start the timer.” And then that person being, like, “Alexa, stop the timer”

and

They’re like, “Oh, whenever I do this, she says this and then that’s a bad thing.”

Other reservations raised by participants were invading privacy with the continuous use of cameras (f=11) and a general discomfort being continuously observed; 1 participant shared the following:

This is crazy, you know, like you’re listening to our conversations, kind of, you know, invading our privacy in our personal space.

Although some participants noted the benefits of alerts, others indicated that many reminders might become annoying (f=6), especially when simultaneously overseeing multiple clients. One participant explained this as follows:

Maybe [the alert’s] on for one person and then you clear that one out for another person. And then you clear that one out for another person. And another person. And another person and by the time you finished clearing out, we started all over again.

Finally, although some participants were enthusiastic about using personal mobile devices, others expressed concerns about using personal cell phones for data collection (f=5). One participant noted as follows:

I had personally run into the issue where sometimes my personal life and my job would get too intertwined so...I don’t think I would want some kind of app on my phone all the time.

#### Theme 4. General Risks and Concerns About Technology for Data Collection

In addition to concerns about specific technological features, participants described the risks associated with relying on technology for data collection. They raised concerns that technology might not work properly when needed (f=19), such as, uncharged devices and malfunctioning apps or software. For example, 1 participant described past experiences with shared cellphones.

A lot of times I ran into an issue...where chargers were going missing. It wasn’t being charged. Um, it was dying. That was our form of communication. So, if I needed to communicate an incident, but I had a dead phone, and I’m at a work setting

Another participant described technology malfunctioning and being outdated,

You want to start inputting data and it freezes on you or, you know, it’s not working or whatever you want on an up-to-date computer system.

Other concerns included the risks of technology breaking or being tampered with (f=15) and dependence on a stable internet connection (f=5), particularly when out in the community. One participant explained this as follows:

Whatever [technology] it is shouldn’t be completely dependent upon, like, needing some kind of cellular access because I know sometimes we go out in the community and I don’t have, I don’t have data even if there’s Wi-Fi. Sometimes it’s slow Wi-Fi and I can’t connect.

Participants also expressed concern about technology replacing DSPs and limiting their human interactions (f=5), and concern over the difficulty of learning to adopt technology (f=4). Interestingly, 1 participant mentioned difficulties with technology use among nonnative English speakers:

I also think that [technology] needs to be user-friendly in that not everyone is native English speaking...so maybe instead of words, icons or something, like, to represent some things...

#### Theme 5. Work-Environment Impact on Data Collection and Documentation

Participants described aspects of their work settings that adversely impact data collection and documentation, including lack of dedicated, competent staff that are adequately prepared or motivated to complete direct care responsibilities while also taking quality data (f=11). Furthermore, participants reported lacking the tools and resources needed to complete their job (f=8), including access to technology to help stay organized or communicate with other staff. They noted that being understaffed (f=7) interfered with their ability to complete direct care responsibilities, while also completing data collection and documentation. Participants also reported staying after a shift ends to document data (f=3) and that retrospective data were less likely to be accurate (f=2) because DSPs may not correctly recall what occurred and could be motivated to quickly report data to end their shift. Finally, 1 participant described concerns with the lack of standardized data entry and data interpretation:

You find that we interpret this data differently...even though it’s supposed to be the same across the board...[w]e interpret it differently.

#### Theme 6. Difficulties With Pencil-and-Paper Data Collection

Nearly all our participants who worked with adults with ASD used pencil-and-paper data collection and documentation methods (13/15, 87%). One participant described being in the “Stone Age with recording data.” Specific challenges with pencil-and-paper data collection include difficulty staying organized and on top of data collection, alongside their many direct responsibilities (f=13). For example:

Every staff person might be engaged...trying to de-escalate the situation and then there’s nobody to record the data on paper.

and another explained,

My least favorite part of the job is having to try to track data and interact with them at the same time...You can’t always stop and grab the paper to say, whatever it is that you need to say and still be actively in a moment with a person served.

They indicated that data captured on paper are not always accurate (f=13), and collection forms are not convenient to transport (f=3) on community outings. Participants also described difficulty in finding the necessary client information within the cumbersome paper documentation (f=4). One participant noted as follows:

You know, there’s all this information on them, right? And all these binders...but I just, I didn’t feel like, you know, there was, adequate time really to prepare and, like, going through all these binders and just really, you know, getting familiar with all aspects of, you know, the, the plans regarding each individual.

### Survey

Table 5 provides the usefulness percentages that the participants assigned to the technology features and data entry methods. The features with the highest usefulness percentage across participants included viewing all tasks for a current shift and who is responsible (106/153, 68.8%), viewing all tasks for a client in the current shift (103/153 67.3%), logging a completed task (102/153, 67.1%), viewing all tasks required of you during the current shift (101/153, 66%), and setting reminders for specific tasks (100/153, 65.4%). For the data entry methods, the highest usefulness percentages were for typing on a phone or tablet (103/153, 67.3%), choosing from options on a touch screen (99/151, 65.6%), and typing on a keyboard (100/153, 65.4%).

**Table 5:** Useful technology features and data entry methods (N=153).

Feature		Not useful, n (%)	Maybe useful, n (%)	Probably useful, n (%)	Definitely useful, n (%)	Useful sum^a^, n (%)
**Technology features**						
	View all tasks for current shift and who responsible	21 (13.7)	27 (17.7)	47 (30.7)	59 (38.6)	106 (69.3)
	View all tasks for client	18 (11.7)	32 (20.9)	41 (26.8)	62 (40.5)	103 (67.3)
	Log task completed	22 (14.5)	28 (18.4)	51 (33.6)	51 (33.6)	102 (66.7)
	View all tasks you need to do	26 (17)	26 (17.0)	41 (26.8)	60 (39.2)	101 (66)
	Set reminders	22 (14.4)	31 (20.3)	50 (32.7)	50 (32.7)	100 (65.4)
	Common phrases to write reports	28 (18.3)	30 (19.6)	42 (27.5)	53 (34.6)	95 (62.1)
	Record voice or text comment	22 (14.4)	37 (24.2)	49 (32)	45 (29.4)	94 (61.4)
	Ask for info from person	24 (15.7)	36 (23.5)	47 (30.7)	46 (30.1)	93 (60.8)
	Indicate event occurrence	22 (14.4)	38 (24.8)	36 (23.5)	57 (37.3)	93 (60.8)
	Marks on human body	23 (15.0)	39 (25.5)	38 (24.8)	53 (34.6)	91 (59.5)
	Let person know something	23 (15)	40 (26.1)	38 (24.8)	52 (34)	90 (58.8)
	Create draft report based on notes	29 (19.1)	33 (21.7)	36 (23.7)	54 (35.5)	90 (58.8)
	Spell check and rewording	32 (20.9)	33 (21.6)	39 (25.5)	49 (32)	88 (57.5)
	Ask other staff for help	30 (19.6)	37 (24.2)	40 (26.1)	46 (30.1)	86 (56.2)
	Record video	36 (23.5)	34 (22.2)	41 (26.8)	42 (27.5)	83 (54.2)
**Data entry methods**						
	Typing on phone or tablet	30 (19.6)	20 (13.1)	45 (29.4)	58 (37.9)	103 (67.3)
	Typing on keyboard	24 (15.7)	29 (19.0)	53 (34.6)	47 (30.7)	100 (65.4)
	Choosing from options on a touch screen^b^	24 (15.9)	28 (18.5)	45 (29.8)	54 (35.7)	99 (65.6)
	Pressing a digital clicker to indicate a behavior or event occurred	28 (18.3)	34 (22.2)	42 (27.5)	49 (32.0)	91 (59.5)
	Speaking (can be converted to text that can be edited)^b^	25 (16.6)	38 (25.2)	39 (25.8)	49 (32.5)	88 (58.2)
	Writing with digital pencil^b^	27 (17.9)	46 (30.5)	36 (23.8)	42 (27.8)	78 (51.7)

^a^Useful variable is the sum of Probably Useful and Definitely Useful.

^b^Owing to 2 missing responses, the N value is 151.

When analyzing qualitative responses regarding any concerns with technology to assist with data collection and documentation, the most frequent theme was that participants had no concerns to note (98/121, 90%). Concerns with technology centered on privacy (10/121, 8.3%; eg, HIPAA-compliant platform, data security if device was lost or stolen, and use in the presence of clients), reliability and accuracy of the technology (6/121, 5%; eg, potential for damage to devices or lost data, inaccurate interpretation or transcription of accents), complexity and efficiency of technology use (4/121, 3.3%; staff not technologically savvy, potential to add more time to documentation and take away time from client interactions), and potential for technology failure (3/121, 2.5%; internet outages and device crashing).

When considering the breakdown of features of technology that would be useful by location, the results indicated that participants considered different aspects of technology to be the most useful. See Table 6 for the rank order of features and the usefulness of the data entry methods. See Table 7 for the rank-order correlations of the features by work setting. The clinical setting was the most disparate from the other settings; rank-order correlations from those working in clinical and other settings ranged from a moderate negative correlation (−0.47) to a weak positive correlation (0.08). The rank-order correlations of the data entry methods are presented in Table 8. School was the most distinct from other settings, with weak negative to weak positive correlations (−0.18 to 0.09) for all settings aside from home, with a strong positive correlation between school and home (0.71). The rank-order correlation between participants 35 years and under and those over 35 years was weak, (*r*_13_=0.10; *P*=.36). See Table 9 for the rank order of features and the usefulness of data entry methods by age. The rank-order correlation by median age showed a weak negative correlation (*r*_4_=−0.09; *P*=.44).

**Table 6:** Rank order of technology and methods of entering data usefulness by work setting^a^.

Feature			Community		Residential		Home		Day		School		Clinic
**Technology features**													
	View all tasks for current shift and who responsible	2		12		2		1^b^		5.5		4	
	View all tasks for client	3		1^b^		6		5.5		5.5		13.5	
	Log task completed	4		8		3.5		10		1^b^		5	
	View all tasks you need to do	6		2		1^b^		3		5.5		10.5	
	Set reminders	1^b^		6		3.5		3		9		7.5	
	Common phrases to write reports	7.5		11		12		10		15		1.5^b^	
	Record voice or text comment	10		4		8.5		12		5.5		7.5	
	Ask for info from person	9		10		8.5		7		2		15	
	Indicate event occurrence	7.5		6		8.5		10		5.5		3	
	Marks on human body	12		6		11		3		14		13.5	
	Let person know something	13.5		3		8.5		8		12.5		12	
	Create draft report based on notes	5		13		5		13		10.5		10.5	
	Spell check and rewording	11		9		13		5.5		12.5		7.5	
	Ask other staff for help	13.5		15		14		14		3		1.5^b^	
	Record video	15		14		15		15		10.5		7.5	
**Data entry methods**													
	Typing on phone or tablet	2		2.5		1^b^		1.5^b^		1.5^b^		1.5^b^	
	Typing on keyboard	3		2.5		2		3		3		4.5	
	Choosing from options on a touch screen	1^b^		1^b^		4		4		5.5		1.5^b^	
	Pressing a digital clicker to indicate a behavior or event occurred	4		4		3		6		1.5^b^		4.5	
	Speaking (can be converted to text that can be edited)	5		5		5		1.5^b^		5.5		3	
	Writing with digital pencil	6		6		6		5		4		6	

^a^Responses are rank ordered by the Useful variable (ie, sum of Probably Useful and Definitely Useful).

^b^Indicates top-ranked feature for the designated group.

**Table 7:** Rank-order correlation of technology usefulness by work setting.

	Community	Residential	Home	Day	School
Residential	0.23				
Home	0.72^a,b^	0.49^c,d^			
Day	0.49^c,d^	0.47^c,d^	0.50^b,e^		
School	0.30^c^	0.03	0.31^d^	–0.07	
Clinic	0.05	–0.47^d^	–0.22	–0.33	0.08

^a^Represents a very strong positive correlation (0.7-1.0).

^b^*P*<.01.

^c^Represents a moderate positive correlation (0.3-0.5).

^d^*P*<.05.

^e^Represents a strong positive correlation (0.5-0.7).

**Table 8:** Rank-order correlation of methods of entering data usefulness by work setting.

	Community	Residential	Home	Day	School
Residential	0.99^a,b^				
Home	0.66^c^	0.64^c^			
Day	0.23	0.19	0.32^d^		
School	0.09	0.04	0.71^a^	–0.18	
Clinic	0.79^a,e^	0.72^a^	0.44^d^	0.58^c^	–0.14

^a^Represents a very strong positive correlation (0.7-1.0).

^b^*P*<.01.

^c^Represents a strong positive correlation (0.5-0.7).

^d^Represents a moderate positive correlation (0.3-0.5).

^e^*P*<.05.

**Table 9:** Rank order of technology and methods of entering data usefulness by age. Responses are rank ordered by the Useful variable (ie, sum of Probably Useful and Definitely Useful).

Feature		≤35 years	>35 years
**Technology features**			
	View all tasks for current shift and who responsible	1^a^	5.5
	View all tasks for client	2	7
	Log task completed	4	1^a^
	View all tasks you need to do	6	2.5
	Set reminders	6	4
	Common phrases to write reports		8.5
	Record voice or text comment	9	10.5
	Ask for info from person	3	12.5
	Indicate event occurrence	6	14
	Marks on human body	12	5.5
	Let person know something	11	12.5
	Create draft report based on notes	9	15
	Spell check and rewording	13	8.5
	Ask other staff for help	14	10.5
	Record video	15	2.5
**Data entry methods**			
	Typing on phone or tablet	3	1^a^
	Typing on keyboard	1^a^	5
	Choosing from options on a touch screen	2	3
	Pressing a digital clicker to indicate a behavior or event occurred	4	4
	Speaking (can be converted to text that can be edited)	6	2
	Writing with digital pencil	5	6

^a^Indicates top-ranked feature for the designated group.

## Discussion

### Overview

Both the focus group participants and survey respondents indicated that a wide range of technological features could be useful. However, both also shared concerns about adopting technology during their day-to-day work. Moreover, there was no clear consensus on what features would be most useful across DSPs; rather, DSP age and their work setting influenced their ratings of the usefulness of different technology features.

### Principal Findings From Focus Groups

Participants in our focus groups described experiences consistent with the literature on burnout and high turnover in DSPs who work with adults with ASD, including being short-staffed and overworked, having many simultaneous job responsibilities, and managing challenging behaviors [3,7]. Our participants raised notable concerns about pencil-and-paper data collection, including perceived poor accuracy. This finding is worrisome because timely and accurate data collection and reporting are critical for treatment decision-making, client safety (eg, medication administration), communication across stakeholders (eg, other DSPs, professionals, the family), and funder payment [12]. The concerns expressed by our participants are consistent with those of special education teachers, who face a high burden of manual data entry to accurately identify antecedents and consequences of behavior while simultaneously interacting with students [26].

Although participants described clear disadvantages of pencil-and-paper data collection, most participants reported that this method is still being used by DSPs to support adults with ASD. This data collection method may add to the DSPs’ stress and result in inefficient use of time. For example, DSPs often need to stay beyond scheduled shifts to complete documentation, must sift through binders of documents to locate the required information, and are challenged to recall data at the end of the shift. One DSP even described quickly writing notes about clients onto strips of tape placed on her hand, so that she could later transfer the information to paper documentation. Although there are several digital technologies designed to facilitate data collection and documentation for individuals who support children with ASD, a thorough review of freely accessible or commercially available platforms that are HIPAA compliant for data transmission revealed that there are no such tools specifically marketed for DSPs serving adults with ASD. Although it may be possible to adapt them, the tools designed for ABA providers working with children with ASD cannot fully meet the unique needs of DSPs serving an adult population (eg, multiple clients served by multiple DSPs and daily shift vs individual treatment sessions).

The DSPs in our focus group also indicated that automated video and audio capture and automated graphing of data could be beneficial. In fact, our participants noted that videos might be helpful in evaluating environmental variables that they may not have observed or captured in the moment. In addition, they recognized that digital technology could help facilitate communication across shifts and among different professionals, because many adults with ASD are served by teams of DSPs that transition in and out over days or weeks. Digital alerts and reminders can help facilitate the continuity of care across teams of DSPs.

Although the DSPs in our focus groups were receptive to using technology, they also voiced concerns about privacy surrounding the use of video and audio capture. These concerns could be addressed through technology that supports selective archiving, wherein an individual is required to engage in a specific action (eg, button press) to store captured video and audio [26]. The focus group participants also indicated that they would need portable technology that can be easily activated, devices that prevent client tampering or destruction, voice capture that accounts for different DSP accents and is not sensitive to client activation, data entry that does not depend on internet connectivity, and selective use of alerts to prevent alert fatigue and desensitization. However, DSPs have differing preferences about the use of personal devices, which could potentially be addressed through a solution that provides multiple different configurations.

### Principal Findings From DSP Survey

To help identify specific features that would be most useful to DSPs, a survey asked DSPs to rate the usefulness of the different features of technology and data entry methods discussed during the focus groups. However, most participants considered nearly all features useful, with little differentiation between the top-ranked features and data entry methods. The features that DSPs viewed as the most useful differed by setting and DSP age. Survey respondents working in residential, day, and home settings considered different task views (ie, shift, client, and DSP) most useful. Meanwhile, DSPs who worked in community settings rated setting reminders the highest in terms of usefulness, whereas those who worked in schools rated logging completed tasks as the most useful. Finally, DSPs in clinical settings rated viewing common phrases that could be used to help write reports and asking other staff for help as the most useful; however, asking other staff for help was ranked 14 of 15 across all DSPs. Clients in clinical settings may be more likely to engage in challenging behaviors than those in other settings.

For data entry, there was more uniformity: typing on a phone or tablet to store information was in the first- or second-rank position for DSPs in all settings. That said, choosing from options on a touch screen was prioritized only by those in community, residential, and clinical settings. Different settings may have unique data entry needs based on the structure of the setting and the type of care provided.

Finally, the weak rank-order correlations suggest that different age groups of DSPs may have different preferences. This finding, together with comments that some DSPs are not particularly technologically savvy, suggests that digital technology for DSPs should feature easy-to-adopt components. Moreover, it is important to include a diverse age range of DSPs when pilot testing digital technologies to support their work and evaluating their usability.

### Comparison With Prior Literature

The findings of this study extend the sparse literature on DSPs who work with adults with ASD to include DSPs’ perspectives on how technology can assist in improving efficiency and job satisfaction. Prior literature has documented that DSPs are in high demand [3]; however, stressors such as staffing shortages, a heavy workload, and documentation requirements contribute to a high turnover rate that further exacerbates the stress for the DSPs remaining in the field [7-9]. Our focus group findings support those reported in prior literature that documentation burdens, in addition to staffing shortages, contribute to DSPs feeling overworked and dissatisfied, specifically in the subset of DSPs serving adults with ASD.

In addition, although the literature on DSP stress, burnout, and turnover is well established [7-9], to date, no studies have documented DSPs’ self-reported perspectives on how technology might increase job satisfaction and decrease turnover. This study fills this gap by documenting DSPs’ perspectives on their needs. The addition of qualitative data on DSPs’ perspectives on what contributes to burnout, turnover, and inefficient documentation is critical for developing solutions that could increase job satisfaction and improve the quality of care for the clients they serve.

Our focus group and survey findings suggest that DSPs perceive technology as a helpful solution for increasing documentation efficiency. Prior literature has demonstrated the utility of technology to increase documentation efficiency and decrease workload in other fields (eg, special education [26]); however, to date, no study has examined how technology could assist DSPs in their daily work across settings (eg, residential, community). This study extends the literature by documenting DSPs’ perspectives on the usefulness of different features of technology and data entry methods that have the potential to increase the efficiency of completing daily job responsibilities such as documentation. The finding that participants perceived flexibility and mobility as benefits of using technology for data collection aligns with the mobile technology literature [18,26] and suggests that DSPs working with adults with ASD could benefit from some aspects of data collection technologies developed for providers serving children with ASD if features were adapted to their needs. However, some features prioritized by participants are unique to DSPs serving the adult population with ASD, such as shift-based documentation (eg, tasks to complete and multiple clients assigned), suggesting the need to develop innovative digital technology for this population.

Furthermore, our finding that technology preferences differ by age group of DSPs is consistent with the literature documenting significant differences in DSP behavior by DSP age when serving individuals with intellectual disabilities [39,40]. Together, the current studies set the stage for technology development for DSPs working with adults with ASD, which directly aligns with the New Jersey Council on Developmental Disabilities’ Position Statement on DSPs, suggesting the need to “evaluate and implement the use of technology as an option for support while simultaneously providing relief to the increased demand for support and support workers” [41].

### Limitations

This study has several limitations that warrant mention. Our focus group sample was limited to 15 DSPs working in a single geographic region. However, this sample included DSPs employed in different settings, ranging from a private home with a single resident to a day program at a large agency, as well as DSPs with a wide range of years of employment in the field (<1-20 years), which likely increases the generalizability of our results. Furthermore, the results relied on reports from DSPs about their experiences with data collection and technology use rather than direct observation. Future research should include observations of DSPs to further understand their needs. In addition, we did not measure participants’ familiarity with and comfort with technology, which may have influenced their perceptions of technology use. Future research should consider how these variables impact perceptions and use of newly developed forms of technology.

Meanwhile, to reduce the burden on survey participants, we did not ask them to rank-order features. Instead, we ordered features from the highest to the lowest usefulness percentages across participants. Many participants endorsed multiple features and data collection methods as useful, and the overall usefulness percentages were similar across the multiple response items. Although useful, these data do not provide a clear ranking hierarchy for those interested in developing technological solutions for DSPs. The survey also did not provide a description of how each technology feature might work in practice or address potential concerns; therefore, participants might have had different interpretations of these features, impacting their ratings. Moreover, the survey did not garner a large number of respondents from all work settings; hence, developing setting-specific solutions would require obtaining more input from DSPs who work in each setting. Finally, most participants who reported no concerns with technology may have simply responded as such to complete the survey and access incentive information.

### Implications

To date, to our knowledge, no other study has examined DSPs’ perspectives on the utility of digital technology to facilitate improved work efficiency and job satisfaction when supporting adults with ASD. Given the heterogeneity in the DSP’s usefulness ratings, one important implication is that a comprehensive technology solution will need to be configurable and personalized. Before implementing certain setting-specific features, such as to-do lists and alert capabilities, which were rated as useful by participants, additional needs analysis is warranted on a setting-by-setting basis. Such features should be developed by first designing and then developing a prototype to meet the needs of a specific setting, keeping in mind that additional features will need to be added to enable a solution that is useful and relevant for DSPs in other settings. Digital technology that supports capturing and organizing information needed for the end of shift documentation is likely to be useful across settings, even if the content of the information and format of reports vary by setting.

Using the salient technology features and data collection methods documented in this study, existing tools and technologies designed to assist ABA providers working one-on-one with children in capturing in-the-moment behavioral data during scheduled programing could be adapted or novel digital technology solutions developed to meet the unique needs of DSPs serving multiple adults during shifts in unique settings (eg, residential and vocational). These tools can be used to help increase the accuracy of data collected by DSPs while simultaneously reducing the amount of effort expended on data collection. Reducing the data collection burden could help increase DSPs’ job satisfaction, potentially reducing turnover and increasing the quality of life for their clients.

In contrast, the DSPs noted that there are risks associated with adopting technology-based data collection tools, including potential loss of data or inability to capture new data during technology failures. Moreover, the DSPs’ comments suggest that it is unlikely that any single technology solution will be appropriate for capturing data about all adults with ASD, both because of client characteristics (eg, level of awareness that DSPs are recording data about them) and DSP preferences (eg, types of devices they could comfortably use to capture data while providing support to clients). The preferences of DSPs are critical to consider as they influence the acceptability and utility of a particular solution.

The benefits and risks of incorporating the technology reported by DSPs in this study have important implications for the future development of tools to support DSPs working with adults with ASD. In exploring technological innovations that could support DSPs who work with adults with ASD, it is imperative that we consider solutions that can be disseminated in the real world and integrated with existing practices and needs in the target setting. Tools intended to facilitate documentation must account for how DSPs can collect data in the moment (eg, when managing challenging behavior), in a portable manner (eg, in the community), during their scheduled shifts, and in a way that communicates critical information to multiple parties in an easy-to-understand manner. Moreover, given the differences in features and data collection methods prioritized by age and the recurrent theme that some DSPs are not technologically adept, digital technologies for DSPs should be simple and intuitive and users should be adequately trained. Considering that user acceptability is critical when developing assistive technology, it is especially important for DSPs, given that the National Alliance for Direct Support Professionals requires that DSPs learn and remain current with documentation management systems [25]. In addition, input from not only DSPs but also other stakeholders, such as behavioral supervisors, agency directors, and families, are important to consider, particularly, as other stakeholders are likely heavily involved in the selection of suitable technology and in the use of data collected.

Finally, this line of research could have a profound impact on the health and welfare of several other adults beyond those with ASD. DSPs help care for many populations in need of ancillary support, including elderly individuals, individuals with physical disabilities, and individuals with severe mental illness [1]. The results suggest that the potential benefits of technology for DSPs who work with adults with ASD may be applicable to other DSP groups that experience similar data collection and documentation burdens.

### Conclusions

DSPs play a critical role in the care of adults with ASD. Targeted efforts must be made to mitigate high turnover rates, poor job satisfaction, and the burden of necessary data collection and documentation. This mixed methods study sets the stage for user-centered technology development that can help improve the effectiveness and job satisfaction of DSPs, and ultimately the quality of life of the clients they serve. The results across studies suggest that digital technology has the potential to provide tangible benefits but also highlights the need for a customizable, configurable solution that offers multiple features to meet the diverse needs of DSPs based on both their preferences and the needs specific to the setting in which they work.

Despite reporting that a wide range of technological features could be useful, DSPs across focus groups and survey responses indicated some concerns with adopting technology during their day-to-day work. Future research should identify setting-specific DSP needs and explore barriers to adopting data collection and documentation technologies, such as costs and adaptability in small operations, and consider preventive measures to protect confidentiality, minimize potential device damage, and prevent data loss.

## Abbreviations

ABA: applied behavior analysis
ASD: autism spectrum disorder
DSP: direct support professional
EHR: electronic health record
HIPAA: Health Insurance Portability and Accountability Act
